# Imaging Diagnosis of Crossed Non‐Fused Renal Ectopia With Hydronephrosis in a Child

**DOI:** 10.1002/ccr3.72488

**Published:** 2026-04-09

**Authors:** Adeel Anwaar, Inam Ul Haq, Farooq Hameed, Aymar Akilimali

**Affiliations:** ^1^ Pakistan Kidney and Liver Institute and Research Center Lahore Pakistan; ^2^ Rashid Latif Medical College Lahore Pakistan; ^3^ Mid City Hospital Lahore Pakistan; ^4^ Department of Research Medical Research Circle (MedReC) Goma Republic of the Congo

**Keywords:** case image, hydronephrosis, non‐fused, renal ectopia

## Abstract

Crossed non‐fused renal ectopia should be considered in children with unexplained hydronephrosis. Anatomical malposition does not necessarily indicate obstruction; functional assessment with MAG3 renogram is essential to guide appropriate management and avoid unnecessary extensive surgery.

**Abbreviations**
CT: computed tomography
eGFR: estimated glomerular filtration rate
ERPF: effective renal plasma flow
MAG3: mercaptoacetyltriglycine (technetium‐99 m MAG3 renal scan)
RFTs: renal function tests
RPUG: retrograde pyeloureterography

## 1 Case Presentation

A 12‐year‐old boy from presented to Urology clinic with complaints of lower abdominal pain persisting for 1 year, accompanied by urinary straining. He reported a good urinary stream, complete bladder emptying, and denied dribbling, hematuria, pyuria, or fever. Examination revealed a distended, tender bladder. Initial ultrasonography showed a crossed ectopic left kidney with mild to moderate hydronephrosis.

**FIGURE 1:**
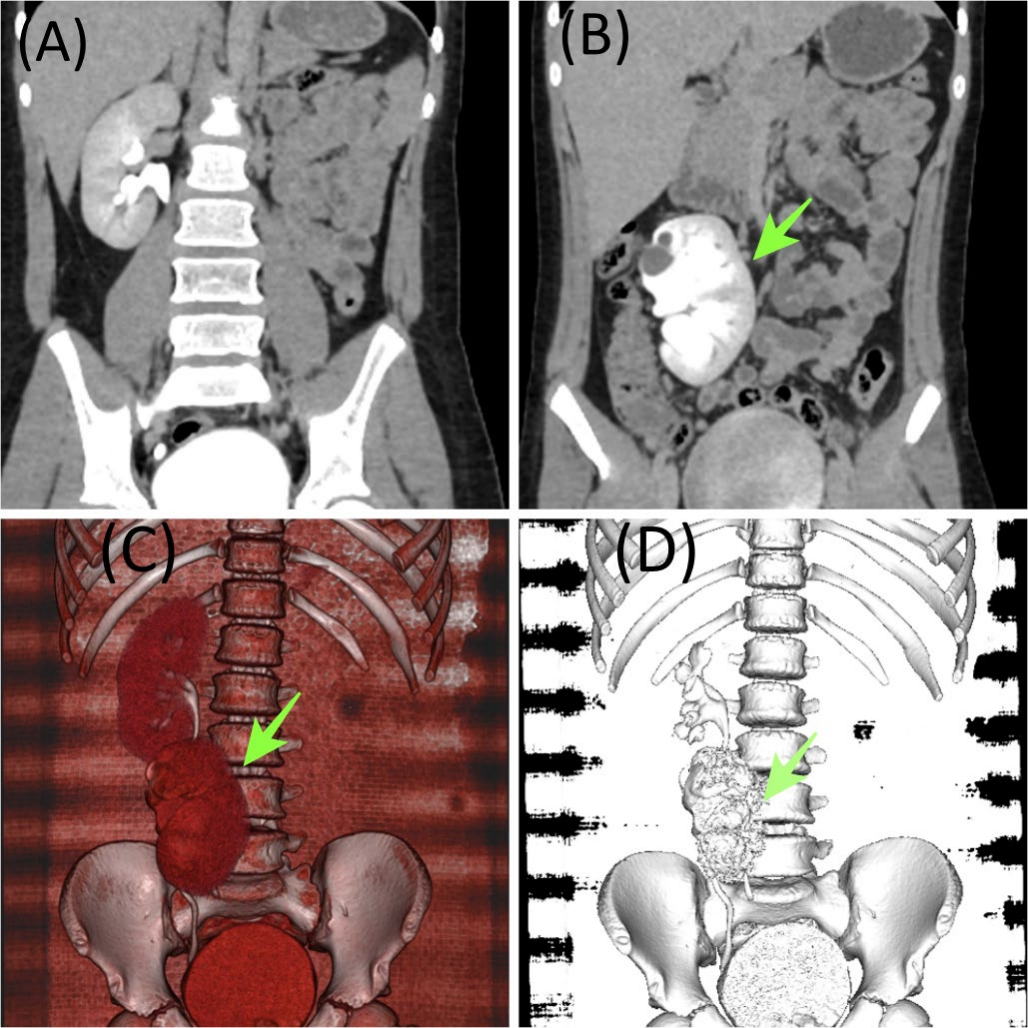
Crossed non‐fused renal ectopia. (A, B) Coronal contrast‐enhanced CT images demonstrating absence of the kidney in its normal anatomical fossa on the left side, with both kidneys located on the same (right) side of the abdomen. The ectopic kidney is positioned inferior to the orthotopic kidney without parenchymal fusion. (C) 3D volume‐rendered reconstruction (anterior view) illustrating two separate renal units on the same side, maintaining distinct renal contours and collecting systems, confirming non‐fusion. (D) 3D skeletal reconstruction highlighting the abnormal renal position relative to the vertebral column and pelvis, with no evidence of renal fusion.

Further evaluation with CT urography demonstrated a mal‐rotated, moderately hydronephrotic left kidney ectopically located in the right pelvic area, just inferior to the normal right kidney, without intervening renal tissue, consistent with crossed non‐fused renal ectopia. Functional imaging (MAG3 renogram) showed equal split renal function (50% each) with diuretic T½ of 9.75 min for the ectopic kidney and 4.62 min for the orthotopic kidney, and an estimated ERPF of 347 mL/min (Figure 1).

Laboratory investigations, including CBC, RFTs, and electrolytes, were within normal limits, with serum creatinine 0.68 mg/dL and eGFR 175.3 mL/min. The patient was deemed fit for surgery. The patient was planned for cystoscopy and retrograde pyeloureterography (RPUG) with possible ureteric reimplantation if narrowing was detected, or double‐J ureteral stent if not. He underwent cystoscopy, RPUG, and double‐J ureteral stenting as narrowing was not detected. Postoperative recovery was uneventful, with hemodynamic stability maintained throughout hospitalization, but symptoms did not settle on follow‐up visits. He was referred to the concerned specialty to look for any other cause.

This case highlights the clinical and surgical management challenges of crossed non‐fused ectopic kidney with hydronephrosis in a pediatric patient having no significant obstruction on MAG3 renogram and RPUG, including the importance of ruling out concurrent acute abdominal pathologies before definitive intervention.

Crossed non‐fused renal ectopia is an exceptionally rare congenital anomaly in which one kidney migrates across the midline to the opposite side of the body but remains anatomically separate from the normally positioned kidney and accounts for only about 10% of cases and has been estimated to occur in 1 in 75,000 autopsies. In crossed ectopia, despite the abnormal position of the kidney, the ureter typically inserts into the bladder at its usual anatomical location [1].

The anomaly is thought to arise from abnormal interaction and migration of the metanephric blastema and ureteric bud during the fourth to eighth weeks of embryonic development. Left‐to‐right crossing is reported more frequently, with a mild male predominance (approximately 1.4–2:1). Although most individuals remain asymptomatic and the condition is often discovered incidentally during imaging or even at autopsy, its clinical importance lies in its association with urinary tract infections, obstruction, stone formation, and hydronephrosis [2].

## Author Contributions

**Adeel Anwaar:** conceptualization, data curation, resources, supervision, writing – original draft, writing – review and editing. **Inam Ul Haq:** conceptualization, data curation, project administration, supervision, writing – original draft, writing – review and editing. **Farooq Hameed:** conceptualization, data curation, project administration, supervision, validation, visualization, writing – original draft, writing – review and editing. **Aymar Akilimali:** conceptualization, data curation, project administration, supervision, validation, visualization, writing – original draft, writing – review and editing.

## Funding

The authors have nothing to report.

## Ethics Statement

This is a case image utilizing anonymized patient information and so was classified as exempt from review from the Institutional Review Board.

## Consent

A written informed consent was obtained from the patient based on the journal's policies.

## Conflicts of Interest

The authors declare no conflicts of interest.

## Data Availability

data is available upon reasonable request.
